# Application of Mathematical Modeling in Prediction of COVID-19 Transmission Dynamics

**DOI:** 10.1007/s13369-021-06419-4

**Published:** 2022-01-07

**Authors:** Ali AlArjani, Md Taufiq Nasseef, Sanaa M. Kamal, B. V. Subba Rao, Mufti Mahmud, Md Sharif Uddin

**Affiliations:** 1 Department of Mechanical & Industrial Engineering, College of Engineering, Prince Sattam Bin Abdulaziz University, AlKharj, 16273 Saudi Arabia; 2 Douglas Hospital Research Center, Department of Psychiatry, School of Medicine, McGill University, Montreal, QC Canada; 3 Department of Internal Medicine, College of medicine, Prince Sattam Bin Abdulaziz University, AlKharj, 11942 Saudi Arabia; 4 Dept of Information Technology, PVP Siddhartha Institute of Technology, Chalasani Nagar, Kanuru, Vijayawada, Andhra Pradesh 520007 India; 5 Department of Computer Science, Nottingham Trent University, Clifton, Nottingham, NG11 8NS UK; 6 Medical Technologies Innovation Facility, Nottingham Trent University, Clifton, Nottingham, NG11 8NS UK; 7 Computing and Informatics Research Centre, Nottingham Trent University, Clifton, Nottingham, NG11 8NS UK; 8 Department of Mechanical & Industrial Engineering, Prince Sattam Bin Abdulaziz University, AlKharj, 16273 Saudi Arabia; 9 Department of Mathematics, Jahangirnagar University, Savar, Dhaka, 1342 Bangladesh

**Keywords:** Coronavirus, ODE, SIR, SEIR, Prediction, SARS-CoV-2

## Abstract

The entire world has been affected by the outbreak of COVID-19 since early 2020. Human carriers are largely the spreaders of this new disease, and it spreads much faster compared to previously identified coronaviruses and other flu viruses. Although vaccines have been invented and released, it will still be a challenge to overcome this disease. To save lives, it is important to better understand how the virus is transmitted from one host to another and how future areas of infection can be predicted. Recently, the second wave of infection has hit multiple countries, and governments have implemented necessary measures to tackle the spread of the virus. We investigated the three phases of COVID-19 research through a selected list of mathematical modeling articles. To take the necessary measures, it is important to understand the transmission dynamics of the disease, and mathematical modeling has been considered a proven technique in predicting such dynamics. To this end, this paper summarizes all the available mathematical models that have been used in predicting the transmission of COVID-19. A total of nine mathematical models have been thoroughly reviewed and characterized in this work, so as to understand the intrinsic properties of each model in predicting disease transmission dynamics. The application of these nine models in predicting COVID-19 transmission dynamics is presented with a case study, along with detailed comparisons of these models. Toward the end of the paper, key behavioral properties of each model, relevant challenges and future directions are discussed.

## Introduction

A large family of viruses (Coronaviruses) that can be a source of disease transmission starts with a typical cold advances to Severe Acute Respiratory Syndrome (SARS). The MERS-coronavirus (Middle East Respiratory Syndrome) was reported in 2012, in the Kingdom of Saudi Arabia (KSA), which generally originated from camel flu and spread as a severe respiratory disorder to humans through various channels [1–3]. The symptoms of respiratory infections lead to an acute form of pneumonia. The current ongoing novel coronavirus disease (COVID-19) outbreaks originated in December 2019 in Wuhan of the Hubei Province, China, and have been linked to the Huanan Wholesale Seafood Market [4–6]. Presently people and the community are suffering due to the (COVID-19) epidemic. Despite the fact that the World Health Organization (WHO) officially declared a pandemic, it spread not only in Asia, but also in Africa, South America, the Middle East and Europe. The panic of the people and their community about the virus (COVID-19) outbreak recalls the London influenza pandemic of 1918. Its mild symptoms in most cases and in short sequential intervals (4-5 days) are identical to those of the influenza pandemic, rather than the severe acute respiratory syndrome coronavirus (SARS-CoV) or the Middle East Respiratory Syndrome coronavirus (MERS-CoV) [7]. As of June 22, 2021, there have been 179,698,836 confirmed cases worldwide with 3,891,374 deaths and 164,394,324 recovered (https://www.worldometers.info/coronavirus/). These numbers are exponentially growing day by day.

At present, people and the community are under threat due to the (COVID-19) disease. Though recently vaccines have been rolled globally, due to the lack of treatment it is challenging for decision-makers to fight against this contagious disease. It is therefore imperative to learn more about the rapid transmission mechanism of the virus and how future measures can be taken to control the spread of infection. Several different epidemiological factors of Severe acute respiratory syndrome coronavirus 2 (SARS-CoV-2) including the reproduction number, are still unknown, as this is a contemporary infectious virus. The basic reproduction number/ratio is a crucial epidemiological factor that is needed to identify the current status of disease outbreaks. Thus, a number of researchers are investigating the high infection rate and transmission patterns following the different characteristics of SARS-CoV-2. Scientists, Doctors and Researchers are all working together to investigate and understand its high infection rate and transmission process [8, 9] along with prevention [10–12] and support [13]. There have been several work involving artificial intelligence and machine learning methods to support COVID-19 [14] through analyzing lung images acquired by means of computed tomography [15], chest X-ray [16–18] and understanding and supporting mental health [19–22]. However, mathematical model is one possible way to understand the basic principle of COVID-19 transmission as well as transmission dynamics and to provide further necessary guidelines for the measures of disease mitigation.

**Fig. 1:**
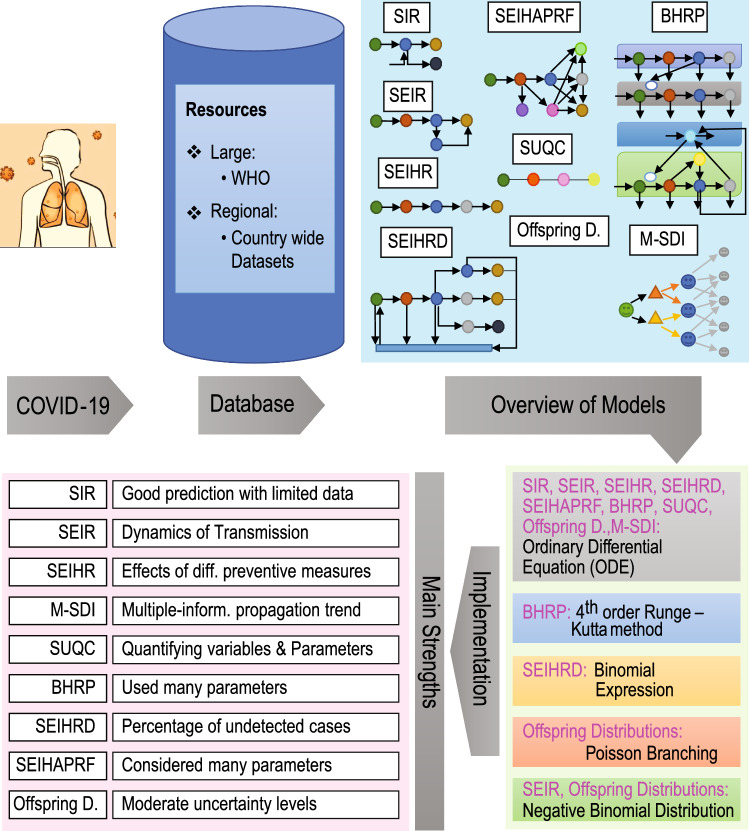
Schematic diagram which includes an illustration of COVID-19 infection, data sources for research, a representative flowchart for each model, the computational mathematics behind it and the main strengths of each model

Mathematical modeling is useful and applicable to assess the sizes, peak and transmission dynamics of a contagious disease such as the novel SARS-CoV-2. For any pandemic of a contagious disease, it is essential to run its affecting parameters into a mathematical testing model to take further measures. There are many mathematical models for infectious diseases, including compartmental models, starting from the classical SIR to more sophisticated models. Such models play an important role in helping to quantify possible infectious disease control and mitigation strategies [23]. Mathematical modeling has been used to analyze multiple characteristics of the disease and can provide the tools to predict the trends of transmission dynamics of a contagious disease such as COVID-19. Mathematical models estimate disease progress that can be helpful for public healthcare interventions and inspecting the momentum of disease outbreaks.

Following studies reported in the literature that have been made available via leading databases (e.g., PubMed (https://pubmed.ncbi.nlm.nih.gov/), IEEEXplore (http://ieeexplore.ieee.org/), Web of Science (https://apps.webofknowledge.com/), Scopus (https://www.scopus.com/)) and Google Scholar (https://scholar.google.com/)) our effort in this review is to study the nine most commonly used models (Fig. 1) based on mathematical implementations and critically review how they play a vital role in investigations of transmission dynamics where the characteristics of contagious diseases are analyzed. The purpose of this review is to provide the following:

- a detailed account of all available mathematical models used in pandemic modeling and prediction;
- a comprehensive survey of state-of-the-art applications of available mathematical models in the modeling and prediction of COVID-19 infection transmission;
- a comparative analysis of different mathematical models on the basis of their usage in COVID-19 infection transmission modeling and prediction;
- an elaborate discussion of open challenges and required future research to fight against the COVID-19 pandemic using mathematical modeling.

First, we divided the total time frame (January 2020–February 2021) of COVID-19 into three phases from the published literature reported in Google Scholar. We run a phase-specific analysis in Sect. 2. The rest of the paper is organized as follows: Sect. 3 provides an account of the available mathematical models, Sect. 4 contains the application details of the mathematical models within the context of COVID-19, including the advantages and disadvantages of each model, Sect. 5 points out some key observable behavior for each of the models within the context of other models, applied methods and result prediction, Sect. 6 provides an overview of other available relevant mathematical models, Sect. 7 describes the upcoming challenges and future research directions, and Sect. 8 presents the discussion and conclusion.

## A Phase-Specific COVID-19 Case Study

In the last year, thousands of articles on COVID-19 were published. For this review, we selected articles that (1) were published in a pure-reviewed journal, (2) performed mathematical analysis, (3) used real data sets for the analysis, (4) were country-specific comparative prediction models and (5) had a high number of citations. We did not consider articles that fell under five above-mentioned issues but were a continuations or extension of a previous similar work (e.g., in terms of country and model).

**Fig. 2:**
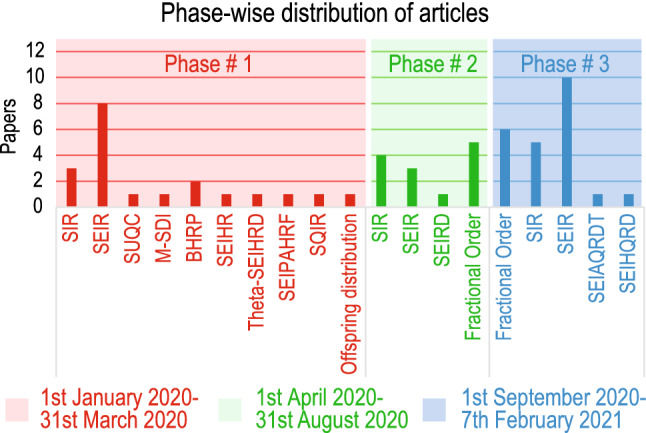
Phase-wise distribution of papers

After carefully investigating the published articles, we divided the total time period of the pandemic into three important phases: Phase #1: initial/rough prediction of disease dynamics (January 1, 2020–March 31, 2020), Phase #2: accurate model development (April 1, 2020–August 31, 2020) and Phase #3: data-driven applications (September 1, 2020–February 6, 2021). For each phase, we prepared a table indicating the publications based on mathematical modeling. The distribution of the selected papers is shown in Fig. 2.

According to our understanding, many simple mathematical models (e.g., SIR and SEIR) were applied with limited datasets on various studies to understand the initial COVID-19 transmission dynamics without proper parameters and validation in Phase #1. Accordingly, many articles with new and complex methods were published following simulation, model development, stabilization and comparisons in Phase #2. Since our focus was to investigate publications based on mathematical models following real data, we list only very few articles on fractional order for method development and simulation studies. Finally, we gradually obtained data-driven complex mathematical modeling to predict more accurate and important information in Phase #3. Many comparative studies and individual complex model articles based on real data were observed in this phase, including fractional order (see Table 1 for more details).

**Table 1:** Phase-wise publications related to mathematical modeling in detecting COVID-19 transmission dynamics

Phase	Model	Ref.	Country/Region/area
Phase#1	SIR	[24]	Mainland China
		[25]	India
		[26]	India
	SEIR	[27]	Wuhan, China
		[28]	Wuhan, China
		[29]	Wuhan, China
		[30]	India
		[31]	Wuhan, Hubei Province and nearby regions
		[32]	Korea
		[33]	Korea
		[7]	Wuhan, China
	SUQC	[34]	Hubei, Wuhan, China
	M-SDI	[35]	China, (data-Chinese Sina-microblog)
	BHRP	[36]	Wuhan City, China
	SEIHR	[37]	Daegu and North Gyeongsang Province, Korea
	Theta-SEIHRD	[38]	Chinese Mainland, Macao, Hong-Kong and Taiwan
	SEIPAHRF	[23]	Wuhan, China
	SQIR	[39]	Pakistan
	Offspring distribution	[40]	Data used from 46 countries reported by WHO
Phase#2	SIR	[41]	China
		[42]	Comparison(china, Italy)
		[23]	Wuhan, China
		[43]	Italy
	SEIR	[44]	The Republic of Kazakhstan
		[45]	India
		[46]	Saudi Arabia
	SEIRD	[47]	London and Wuhan
	FO (DECS)	[48]	N/A (Simulation)
	FO (CFFD)	[49]	N/A (Simulation)
	FO (KTFD)	[50]	N/A (Simulation)
	ASM	[51]	USA, UAE and Algeria
	FO (CS)	[52]	N/A (Simulation)
Phase#3	Fractional order	[53]	Wuhan, China
		[54]	Simulation, Wuhan, China
		[55]	Saudi Arabia
		[56]	USA
		[57]	Nigeria
		[58]	Pakistan
	SIR	[59]	Comparative study (China, South Korea, India, Australia, USA, Italy)
		[60]	WHO data
		[61]	Brazil
		[62]	Brazil
		[63]	Malaysia
	SEIR	[64]	Pakistan
		[65]	USA
		[66]	Morocco
		[67]	Simulation, India
		[68]	Saudi Arabia
		[69]	Egypt & Oman
		[70]	Saudi Arabia
		[71]	India
		[72]	Comparative (China, South Korea, Italy and Iran)
		[73]	China
	SEIAQRDT	[74]	India
	SEIHQRD	[75]	Kenya

*Ref*: reference; *FO (DECS)*: fractional order (differential equations in the Caputo sense); *FO (CFFD)*: fractional order (Caputo-Fabrizio fractional derivative); *FO (KTFD)*: fractional order (kernel type of fractional derivative); *FO (CS)*: Fractional Order in the Caputo sense; *ASM*: age-structured model (based on differential equations)

## Overview of Mathematical Models used in Pandemic Modeling

To carefully evaluate the available mathematical models used in pandemic modeling and prediction, we searched the leading databases of academic literature with specific search strings containing the following keywords: ‘mathematical modeling’, ‘COVID-19’, ‘coronavirus’, ‘corona’, ‘SARS-CoV-2’, ‘transmission dynamics’ and ‘infection transmission dynamics’. The databases used were PubMed, IEEEXplore, Web of Science and Scopus. The search generated a total of 193 articles (PubMed: 20; IEEEXplore: 35; Scopus: 34; Web of Science: 104), which were then manually scrutinized by removing duplicates, checking for relevance and removing papers published before the onset of the disease. This resulted in 21 articles that used nine different mathematical models to model and predict COVID-19 infection transmission dynamics.

This section focuses on the nine selected contagious disease models with mathematical analysis implementation and model diagrams in the context of the infection transmission dynamics of the novel SARS-CoV-2 disease. These nine selected models are also used for further investigation in Sects. 4 and 5.

### Model 1: Susceptible-Infectious-Removed (SIR)

**Fig. 3:**
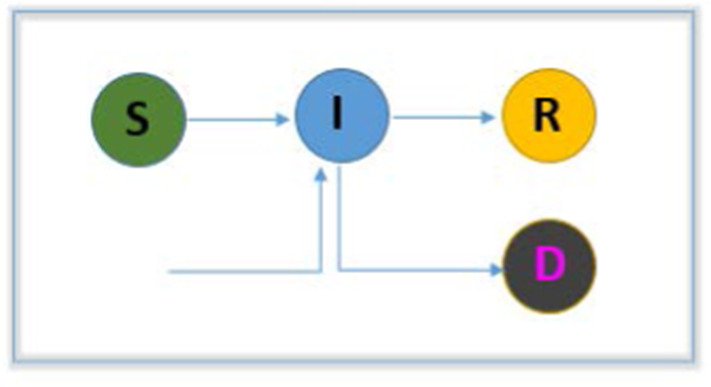
A flowchart representation of SIR model; [Susceptible (S)-Infectious (I)-Removed (R)]

The simplest compartmental model is SIR (‘Susceptible-Infectious-Removed’) where S, I and R are the susceptible, infectious (can infect others) and removed (recovered or dead) [76] populations, respectively. A flowchart of this model is presented in Fig. 3. There are many models that have been used to investigate the transmission dynamics of viruses, and those models are in some way derivatives of the basic SIR model. For the present pandemic circumstances, many researchers have developed SIR-based mathematical models. The model is formulated by a set of nonlinear ordinary differential equations (ODEs) and then solved numerically. The simplest form of nonlinear ODEs can be expressed as in Eq. 1.

1 $$\begin{aligned} \frac{\mathrm{d}}{\mathrm{d}t}(S)&=-\beta SI\nonumber \\ \frac{\mathrm{d}}{\mathrm{d}t}(I)&=\beta SI - \alpha I\nonumber \\ \frac{\mathrm{d}}{\mathrm{d}t}(R)&=\alpha I \end{aligned}$$

(Parameters: $\alpha$—removal rate, $\beta$—infection rate).

In this study [25], an age-structured SIR mathematical model considering social connection matrices based on surveys and Bayesian imputation is presented to inspect the momentum of the SARS-CoV-2 pandemic in India. This study accentuated the importance of both social contact and age structures in appraising the country-specific impacts of the widely used social distancing strategy for controlling and mitigating the virus. In [24], the authors proposed a simplified SIR mathematical model to predict the peak of the disease infection and suggested that the healthcare system could significantly shorten the outbreak period and reduce one-half of the transmission. In another study, [41], an SIR model is used to predict disease (COVID-19) trends and how quarantine decreases infection.

### Model 2: Susceptible-Exposed-Infectious-Removed (SEIR)

**Fig. 4:**
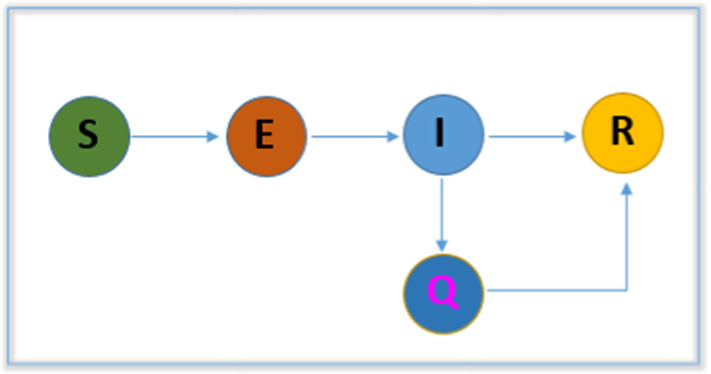
A flowchart representation of SEIR model; [Susceptible (S)-Exposed (E)-Infectious (I)-Removed (R)]

A compartmental model SEIR (‘Susceptible-Exposed-Infectious-Removed’) with S, E, I and R represents the Susceptible, Exposed (infected, but not infectious), Infectious (can infect others) and Removed (recovered or dead) populations. A flowchart of this model is presented in Fig. 4.This model significantly simplifies the mathematical modeling of different infectious diseases such as the novel SARS-CoV-2. Thus, the model is formulated by a set of nonlinear ordinary differential equations (ODEs) and therefore solved numerically. The simplest form of a set of ODEs for the SEIR-based model is as follows:

2 $$\begin{aligned} \frac{\mathrm{d}}{\mathrm{d}t}(S)&=-\beta \frac{I}{N}S-\beta _{F}(1-e^{-\tau C})S\nonumber \\ \frac{\mathrm{d}}{\mathrm{d}t}(E)&=\beta \frac{I}{N}S+\delta \beta \frac{I}{N}S-\kappa E\nonumber \\ \frac{\mathrm{d}}{\mathrm{d}t}(I)&=\kappa E-\alpha I\nonumber \\ \frac{\mathrm{d}}{\mathrm{d}t}(R)&=\gamma C \end{aligned}$$

(Parameters and Notations: $\beta _{F}$—Behavior change transmission rate, $\beta$—Transmission rate between two groups, $\tau$—Scaling factor, $\delta$—Transmission reduction component, $\kappa$—Progression rate, $\alpha$—Confirmation rate, $\gamma$—removal rate, *C*—Confirmed and isolated, *N*—Population)

In the current pandemic, many researchers are adopting the SEIR model to determine the transmission dynamics of COVID-19. Accordingly using the SEIR model, in [32], the authors described transmission dynamics by quantifying the school closure potential effect on the disease and mainly investigated child-to-child infection transmission. In their other work [33], they predicted the pattern of local transmission dynamics based on changes in individuals’ behavior in Korea, and they found a per-capita infection transmission rate that was 8.9 times higher in the local area (Daegu/Gyeongbuk) than nationwide (average). Likewise, the authors of [7, 27–31, 35, 77–79] proposed and formulated the model with a set of ODEs considering different transmission pathways and symptoms. They identified transmission dynamics of the virus and suggested different control measures. Earlier, in [80], the authors formulated a SEIRS model for avian influenza that includes bird human interaction by investigating the essential transmission dynamics of the disease based on equilibrium analysis.

**Fig. 5:**
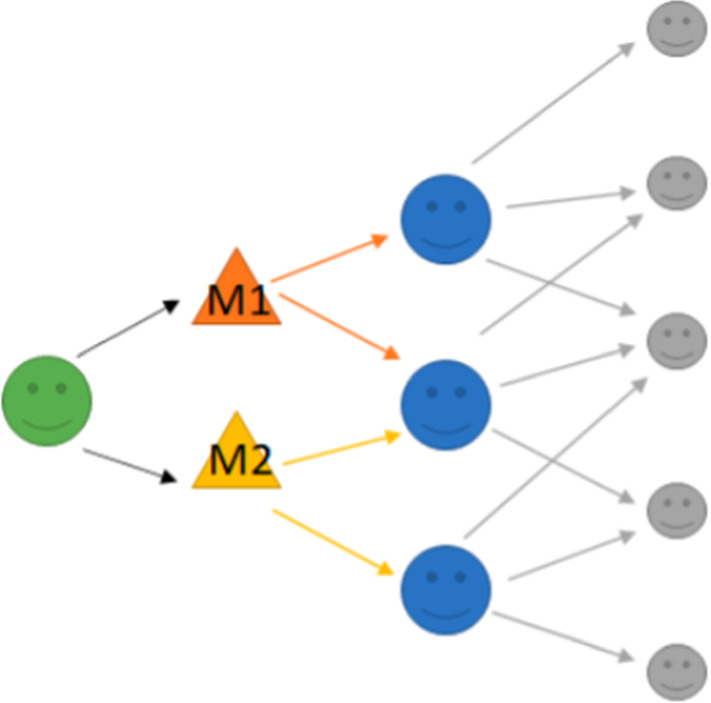
Flowchart representation of the M-SDI model; [multiple (M)-information-susceptible (S)-discussing (D)-immune (I)]

### Model 3: Multiple-Information Susceptible-Discussing-Immune (M-SDI)

The M-SDI (multiple-information susceptible-discussing-immune) dynamic model proposed in [35] was used to understand the types of significant information propagation on social media based on the amount of public discussion and the frequent behavior change (searches/comments) of users. A sample flowchart of this model is presented in Fig. 5. These authors estimated reproduction ratio decreases from 1.7769 to approximately 0.97, which means that the public discussion peak has passed, but it will still progress for a period of time. The model is illustrated mathematically as follows:

3 $$\begin{aligned} \frac{\mathrm{d}}{\mathrm{d}t}(S)&=-\beta SD+\left( 1-p-q \right) \beta SD+\theta \alpha D\nonumber \\ \frac{\mathrm{d}}{\mathrm{d}t}(D)&=p\beta SD-\alpha D\nonumber \\ \frac{\mathrm{d}}{\mathrm{d}t}(I)&=q\beta SD+(1-\theta )\alpha D \end{aligned}$$

To explore some of the characteristics of the qualitative nature of the prediction in the M-SDI model, authors have used the LS method for estimating parameters and primary susceptible population. Accordingly they estimated parameters in their model with data of at least 3–4 days’ to predict public discussion trends at different phases.

### Model 4: Susceptible-Unquarantined Infected-Quarantine Infected-Confirmed Infected (SUQC)

**Fig. 6:**
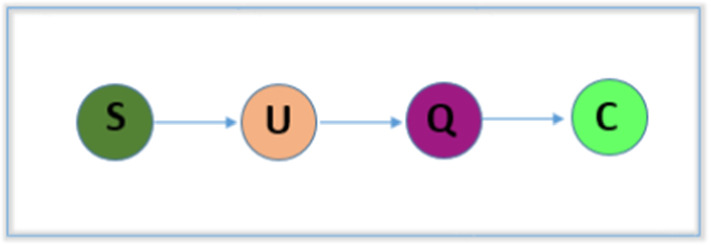
Flowchart prepresentation of the SUQC model; [susceptible (S)-unquarantined (U)-infected quarantine (Q)-infected-confirmed (C)-infected]

SUQC (‘susceptible-unquarantined infected-quarantine infected-confirmed infected’) is a compartmental model where S, U, Q and C represent the susceptible (S is similar as in the existing infectious virus transmission models SIR and SEIR), un-quarantined infected (infected and un-quarantined cases different from E in the existing SEIR model), quarantine infected (quarantine infected cases) and confirmed infected population. A flowchart of this model is presented in Fig. 6. Essentially, in [34], the authors developed the SUQC model to describe the transmission dynamics of the novel SARS-CoV-2 and especially the interference effects of the control measures by analyzing the disease outbreak. The method is formulated with a set of ODEs as follows:

4 $$\begin{aligned} \frac{\mathrm{d}}{\mathrm{d}t}(S)&=-\alpha \frac{S}{N}U\nonumber \\ \frac{\mathrm{d}}{\mathrm{d}t}(U)&=\alpha \frac{S}{N}U-U(\gamma +(1-\gamma )\delta )\nonumber \\ \frac{\mathrm{d}}{\mathrm{d}t}(Q)&=\gamma U-\beta Q\nonumber \\ \frac{\mathrm{d}}{\mathrm{d}t}(C)&=\beta Q+(1-\gamma )\delta U \end{aligned}$$

(Parameters: $\alpha$—Infection rate, $\gamma$—Quarantine rate, and $\beta$—Total confirmation rate.)

This model is adapted to the data of daily released confirmed cases to analyze the outbreaks of the diseases in Hubei, Wuhan, and four other first-tier cities in China. Authors have demonstrated authentic predictions of the transmission trends considering multiple characteristics, which include high infectivity, time delay and intervention effects. However, SUQC can quantify ariables and parameters regarding the intervention effects of the outbreaks. According to the simulation results the method further provides guidance in controlling disease spread.

**Fig. 7:**
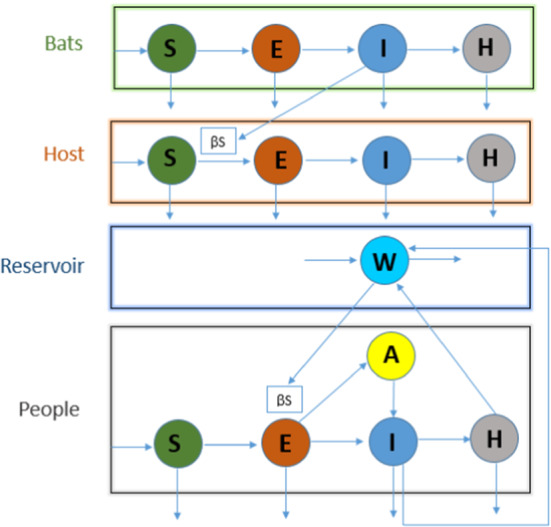
Flowchart representation of the BHRP model; [Bats (B)-Hosts (H)-Reservoir (R)-People (P); Susceptible (S)-Exposed (E)-Infectious (I)-Removed (R)]

### Model 5: Bats-Hosts-Reservoir-People (BHRP)

BHRP (Bats-Hosts-Reservoir-People) is a network model for simulating the transmission of the virus, where B, H, R and P represent the bats (probable infection source), hosts (unknown but probably wild animals), reservoir (seafood market) and people (exposed population) developed in [36]. A flowchart of this model is presented in Fig. 7. In this method, the authors ignored the Bats-Host transmission network and presented the BHRP model in a simplified RP (Reservoir-People) model. They divided people into five different compartments: susceptible, exposed, symptomatic infected, asymptomatic infected and removed. The simplified model is illustrated mathematically as follows:

5 $$\begin{aligned} \frac{\mathrm{d}}{\mathrm{d}t}(S_{p})&=\mu _{p}-(I_{p}+\sigma A_{p})y_{p}S_{p}-\beta _{p}S_{p}X \nonumber \\ \frac{\mathrm{d}}{\mathrm{d}t}(S_{p})&=(I_{p}+\sigma A_{p})\beta _{p}S_{p}+\beta _{x}S_{p}X-(1-S_{p})\omega _{p}E_{p}\nonumber \\&~~~~~-S_{p}\omega _{p}^{'}E_{p}-y_{p}E_{p} \nonumber \\ \frac{\mathrm{d}}{\mathrm{d}t}(I_{p})&=(1-\delta _{p})\omega _{p}E_{p}-(\gamma _{p}+y _{p})I_{p} \nonumber \\ \frac{d}{dt}(A_{p})&=S_{p}\omega _{p}^{'}E_{p}-(\gamma _{p}^{'}+y_{p})R_{p} \nonumber \\ \frac{\mathrm{d}}{\mathrm{d}t}(R_{p})&=\gamma _{p}I_{p}-\gamma _{p}^{'}A_{p}-y_{p}R_{p} \nonumber \\ \frac{d}{dt}(X)&=\rho _{p}I_{p}+\rho _{p}^{'}A_{p}-\varepsilon X \end{aligned}$$

($y_{p}$—death rate (people), $\sigma$—multiple of transmissibility ($A_{p}$ to $I_{p}$), $\delta _{p}$—infection rate (asymptomatic people), $\beta _{p}$—transmission rate ($I_{p}$ to $S_{p}$))

They estimated the basic reproduction number according to their numerical illustration by assessing the transmissibility of the virus based on a simplified RP model, and they found values of 2.30 (reservoir-person) and 3.58 (person-person). The results showed that the transmissibility of SARS-CoV-2 is higher than that of the MERS in the Middle East and is similar to that of serious respiratory syndrome. They also found that the transmissibility is smaller than that of MERS in Korea.

**Fig. 8:**
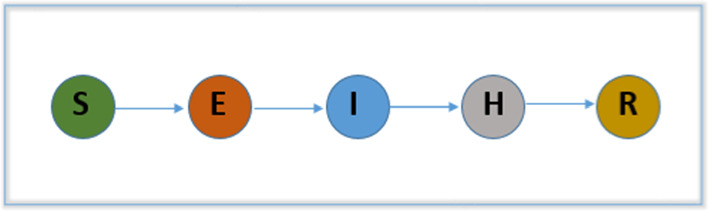
Flowchart representation of the SEIHR model; [Susceptible (S)-Exposed (E)-Symptomatic infectious (I)-Hospitalized (H)-Removed (R)]

### Model 6: Susceptible-Exposed-Symptomatic infectious-Hospitalized-Removed (SEIHR)

SEIHR (Susceptible-Exposed-Symptomatic infectious-Hospitalized-Removed) is a virus transmission deterministic model where S, E, I, H and R represent the susceptible, exposed, symptomatic infectious, hospitalized and removed (recovered or death) populations. A flowchart of this model is presented in Fig. 8. With this model, in [37], the authors estimated the size of the outbreak and the reproduction number and found that, if the rate of transmission decreases, the outbreak of the disease ends early and the virus infection cases also decrease. The mathematical implementation of the model is illustrated with a system of nonlinear ODEs as follows:

6 $$\begin{aligned} \frac{\mathrm{d}}{\mathrm{d}t}(S)&=-\beta \frac{SI}{N} \nonumber \\ \frac{\mathrm{d}}{\mathrm{d}t}(E)&=\beta \frac{SI}{N}-\sigma E \nonumber \\ \frac{\mathrm{d}}{\mathrm{d}t}(I)&=\sigma E-\alpha I \nonumber \\ \frac{\mathrm{d}}{\mathrm{d}t}(H)&=\alpha I-\gamma H \nonumber \\ \frac{\mathrm{d}}{\mathrm{d}t}(R)&=\gamma H \end{aligned}$$

($\beta$—Transmission rate, $\sigma$—Progression rate, $\alpha$—Isolation rate, $\gamma$—Removal rate).

In the study the authors did not consider natural deaths and births, infections during latency, asymptomatic infections, or re-infected cases. However, according to their simulation results, they suggested different social awareness activities such as wearing masks and social distancing, to reduce the fast transmission of the virus.

### Model 7: Susceptible-Exposed-Infectious-Hospitalized- Recovered-Dead (*Theta*-SEIHRD)

**Fig. 9:**
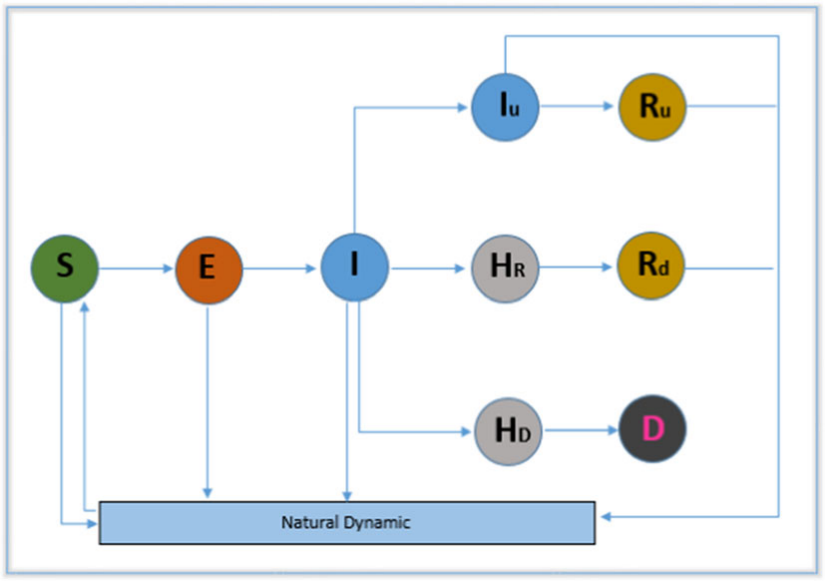
Flowchart representation of the *Theta*-SEIHRD model; [Susceptible (S)-Exposed (E)-Symptomatic infectious (I)-Hospitalized (H)-Removed (R)-Dead (D)]

An extended compartmental model *Theta*-SEIHRD (Susceptible-Exposed-Infectious-Hospitalized-Recovered-Dead) where S, E, I, H, R and D represent the susceptible, exposed (incubation period), infectious (undetected but still can infect others), hospitalized (recovered or dead), recovered (previously detected and previously undetected but infectious) and dead populations, was developed in [38]. A flowchart of this model is presented in Fig. 9. The model is illustrated mathematically as follows (presented concisely here for simplicity):

7 $$ \begin{aligned} \frac{\mathrm{d}}{\mathrm{d}t}(S)&=-\frac{S}{N}(m_{\varepsilon }\beta _{\varepsilon }E+m_{i}\beta _{i}I+m_{iu}\beta _{iu}\theta I_{u})\nonumber \\ {}&~~~ -\frac{S}{N}(m_{hr}\beta _{hr}H_{r}+m_{hd}\beta _{hd}) \nonumber \\ \frac{\mathrm{d}}{\mathrm{d}t}(E)&=\frac{S}{N}(m_{\varepsilon }\beta _{\varepsilon }E+m_{i}\beta _{i}I+m_{iu}\beta _{iu}\theta I_{u})\nonumber \\ {}&~~~+(m_{hr}\beta _{hr}H_{r}+m_{hd}\beta _{hd})-\gamma _{e}E \nonumber \\ \frac{\mathrm{d}}{\mathrm{d}t}(I)&=\gamma E-\gamma I \nonumber \\ \frac{\mathrm{d}}{\mathrm{d}t}(H)&=\gamma I-\gamma H \nonumber \\ \frac{\mathrm{d}}{\mathrm{d}t}(R)&=\gamma H (\mathrm{infection\ detected\  \& \ infection\ undetected}) \nonumber \\ \frac{\mathrm{d}}{\mathrm{d}t}(D)&=\gamma H(\mathrm{Dead \ Compartment}) \end{aligned}$$

(Parameters: $\gamma$—Transition rate for different compartment, $\beta$—disease contact rate for different compartment $\theta$—fraction of infected people).

This model investigates the transmission dynamics of the disease considering the known major characteristics of the disease as the presence of infectious undiscovered as well as detected cases and the distinct sanitary and infectiousness conditions of hospitalized individuals. This model is able to estimate the required number of beds needed in hospitals, and it can calculate the basic reproduction number.

**Fig. 10:**
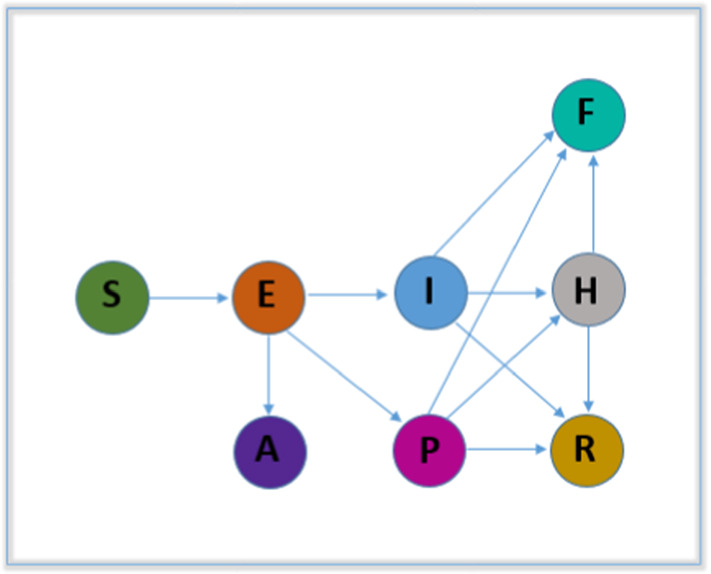
Flowchart of the SEIPAHRF model; [susceptible(S)-exposed(E)-symptomatic and infectious(I)-super spreaders(P)-infectious but asymptomatic(A)-hospitalized(H)-recovery(R)-fatality (F)]

### Model 8: Susceptible class-Exposed class-Symptomatic and Infectious class-Super spreaders class-Infectious but Asymptomatic class-Hospitalized class-Recovery class-Fatality class(SEIPAHRF)

A compartmental model SEIPAHRF (susceptible class-exposed class- symptomatic and infectious class-super spreaders class-infectious but asymptomatic class- hospitalized class-recovery class-fatality class) divided into eight epidemiological compartment where S, E, I, P, H, R and F represent the susceptible individuals, exposed, symptomatic and infectious, super-spreaders, infectious but asymptomatic, hospitalized, recovered and dead populations, was developed in [23]. A flowchart of this model is presented in Fig. 10. The model is illustrated mathematically with a system of nonlinear ODEs as follows:

8 $$\begin{aligned} \frac{d}{dt}(S)&=-\beta \frac{SI}{N}-m\beta \frac{SH}{N}-\beta ^{'}P\frac{S}{N} \nonumber \\ \frac{d}{dt}(E)&=\beta \frac{SI}{N}+m\beta \frac{SH}{N}+\beta ^{'}P\frac{S}{N}-\sigma E\nonumber \\ \frac{d}{dt}(I)&=\sigma \rho _{1}E-(\gamma _{a}+\gamma _{b})I-\delta _{b}I \nonumber \\ \frac{d}{dt}(P)&=\sigma \rho _{2}E-(\gamma _{a}+\gamma _{b})P-\delta _{p}P \nonumber \\ \frac{d}{dt}(A)&=\sigma (1-\rho _{1}-\rho _{2})E \nonumber \\ \frac{d}{dt}(H)&=\gamma _{a}(I+P)-\gamma _{c}H-\delta _{h}H\nonumber \\ \frac{d}{dt}(R)&=\gamma _{b}(I+P)+\gamma _{c}H \nonumber \\ \frac{d}{dt}(F)&=\delta _{b}I+\delta _{p}P+\delta _{h}H \end{aligned}$$

($\beta$—transmission coefficient (infected individuals), $\beta '$—transmission coefficient (super-spreaders), *m*—Relative transmissibility (hospitalized patients), $\sigma$—infectious rate (from exposed people), $\rho _{1}$—Rate (exposed individual become infected *I*) $\rho _{2}$—Rate (exposed individual become super-spreaders), $\gamma _{a}$—Rate (hospitalized), $\gamma _{b}$—Recovery rate (without hospitalized), $\gamma _{c}$—Recovery rate (hospitalized patients), $\delta _{b}$—Death rate (infected class), $\delta _{p}$—Death rate (super-spreaders class), $\delta _{h}$—Death rate (hospitalized class))

In [23], the authors (F Ndairou and colleagues) studied the stability of the free equilibrium of the basic reproduction number and investigated the sensitivity by considering the variation in its parameters. They analyzed and simulated the current outbreak considering some important aspects of virus transmission and gave an acceptable approximation based on the data in Wuhan, China. They also fitted the model with daily confirmed cases. According to their findings, the numerical results reflect the real scenario of the Wuhan outbreak.

### Model 9: Offspring Distribution

To quantify the individual human-to-human variation of COVID-19 transmission and observe the outbreak sizes in affected countries, in [40], the authors proposed an interesting mathematical model applying a branching process model where the number of secondary transmissions was assumed to follow a negative binomial distribution.

By assuming that the secondary transmissions (offspring distributions) of COVID-19 cases were independently and identically distributed in a negative binomial distributions. The authors calculated likelihood following a previous study [81]. They computed the probability mass function for a final cluster size resulting from *s* initial cases following the formula below:

9 $$\begin{aligned} c(x;s)&=P(X=x;s)\nonumber \\&=\frac{ks}{kx+x-s}\left( {\begin{array}{c}kx+x-s\\ x-s\end{array}}\right) \frac{(\frac{R_{0}}{k})^{x-s}}{(1+\frac{R_{0}}{k})^{kx+x-s}} \end{aligned}$$

Adjusting the future growing issue for cluster size, the research team introduced a corresponding likelihood functions presented below:

10 $$\begin{aligned} c_{0}(x;s)=P(X\ge x;s)=1-\sum _{m=0}^{x-1}c(m;s) \end{aligned}$$

To compute the total likelihood, the research group applied a final likelihood cluster size of certain countries with other ongoing outbreak countries following the formula below:

11 $$\begin{aligned} L(R_{0}, K)=\prod _{i\epsilon A}^{}P(X=x_{i};s_{i})\prod _{i\epsilon B}^{}P(X\geqslant x_{i};s_{i}) \end{aligned}$$

For statistical analysis, they used a Markov chain Monte Carlo (MCMC) method with 95% credible intervals (CrIs). To determine the best method, they compared negative-binomial branching process model with a Poisson branching process model. However, they used simulations to investigate potential bias caused by under-reporting ( failure to fully report), one of the major limitations of their study.

## The State-of-the-Art Application of Mathematical Modeling: A COVID-19 Case Study

### Model 1: Susceptible-Infectious-Removed (SIR)

To estimate the epidemic size, predict disease transmission trends and forecast the advancement of the disease, the basic SIR model (Fig. 3) was applied to the present COVID-19 pandemic to understand the effect of interventions such as quarantine, social distancing on disease spread and to control and mitigate virus outbreaks. We found three different studies [24, 25, 38] based on the basic SIR mathematical model. In [38], the authors used an SIR model to predict disease (COVID-19) trends and the extent to which quarantine decreased infection. In [24], the authors proposed a simplified SIR mathematical model to predict the peak of disease infection. In [25], an age-structured SIR mathematical model considering social connection matrices based on surveys and Bayesian imputation is presented to determine the momentum of the SARS-CoV-2 pandemic in India. According to the simulation results these studies accentuated the importance of the strategy of social distancing, improvement of the healthcare system, age-structuring and other intervention measures for controlling and mitigating COVID-19 outbreaks. To prevent the rise of disease infection they evaluated different policies in the well-defined contexts of COVID-19. In the presented models some of the parameters and variables were estimated, and some were composed from other published articles. To formulate the models and numerical illustrations they used a set of nonlinear ordinary differential equations (ODEs) (for more details see Overview section). In mathematical simulation reasonable data availability for better estimation and prediction was a challenge. In most cases, they used short and limited epidemiological data, and in some cases, unreasonable released data. As their main objectives were to identify the impacts of lockdowns/ quarantines, infection rates and social distancing through parameter estimation, these models were suggested for disease mitigation policies according to their numerical illustrations.

### Model 2: Susceptible-Exposed-Infectious-Removed (SEIR)

The SEIR is a simple compartmental model and different from SIR; in the SEIR model (Fig. 4) the exposed compartment and a parameter (the movement from the exposed compartment to the infected compartment) are added to describe the transmission dynamics of exposed individuals. This framework significantly simplifies the mathematical modeling of the present novel SARS-CoV-2 pandemic. Recently, several studies have focused on mathematical modeling and have adopted the SEIR model with a mathematical implementation (for more details see Overview section) to estimate transmission and to predict the trends of COVID-19 outbreaks. In their studies, they considered multiple transmission pathway mechanisms, including environment-to-human and human-to-human routes, in the infection dynamics, emphasizing the role of the environmental source in transmission and growth. These studies applied nonlinear ODEs and MCMC methods to calculate the basic reproduction number. This parameter thus provides valuable data on of the current COVID-19 outbreaks.

### Model 3: Multiple-Information Susceptible-Discussing-Immune (M-SDI)

The M-SDI (Fig. 5) was developed to understand the types of significant information propagation on social media based on the amount of public discussion and considering the frequent behavior change (searches/comments) of users. In this work, they focused on the characteristic of users who choose to re-enter related information after they finish a discussion about certain topics. Based on analysis of a model simulation of the multiple-information generation mechanism regarding COVID-19, this model can distinctly predict the evolution of public opinion. They estimated that the reproduction ratio decreases from 1.7769 to approximately 0.97, which means that the public discussion peak was passed, but it will still progress for a period of time. To explore the qualitative nature of prediction in the M-SDI model (Figure 5), the authors used the LS method for estimating parameters and the primary susceptible population. Accordingly, they also estimated parameters in their model with the data of at least 3 to 4 days to predict public discussion trends at earlier phases. The M-SDI model can predict multiple-information development trend during a large-scale community health emergency. The authors used limited data for the estimation of parameters from the most popular Chinese Sina-microblog.

### Model 4: Susceptible-Unquarantined Infected-Quarantine Infected-Confirmed Infected (SUQC)

The SUQC model (Fig. 6) was developed to characterize the transmission dynamics of the novel SARS-CoV-2 and especially parameterize the effects of interventions measures. SUQC is not the same as SEIR, as infected people are classified into confirmed, quarantined and un-quarantined in SUQC. Moreover, in this case, un-quarantined people are able to infect susceptible people only, but in SEIR, infected individuals are infectious. Additionally, the quarantine rate in SUQC is used especially to model the influence of quarantine and other control measures. According to the author’s explanation, the model is more effective than other available epidemic models for analyzing the dynamics of the disease. This model was fitted with daily released confirmed cases data to analyze the outbreaks patterns of the diseases in Hubei, Wuhan, and four other first-tier cities in China. This model employs deterministic ODEs and it was needed to interpret certain uncertainties in this case. The authors demonstrated an authentic prediction of transmission trends considering multiple characteristics including high infectivity, time delay and interventions effects. However, SUQC can quantify variables and parameters regarding the intervention effects of the outbreaks. According to the simulation results, they found a reproduction of number greater than 1 before Jan 30, 2020, in Hubei, Wuhan, and other first-tier cities in China except Beijing, and after Jan 30, 2020, they found a reproduction of number less than 1, in all regions, indicating the effectiveness of the control measures. Subsequently the method further provides guidance controlling disease spread.

### Model 5: Bats-Hosts-Reservoir-People (BHRP)

The Reservoir-People (RP) transmission model (Fig. 7) was developed in [36], considering the routes from reservoir (market) to person and from person to person for severe acute respiratory syndrome SARS-CoV-2. Published data of Wuhan City, China, were used to fit the model. The simulation results found basic reproduction numbers for SARS-CoV-2 of 3.58 from person to person and of 2.30 from reservoir to person. This model might predict interesting transmission chains applying limited data involving a number of important parameters. However, these predictions might not reflect the actual situation of the early stage virus transmission because some parameters were not taken from the accurate database or were applied through assumption.

Overall, the objective of this study was to provide an effective mathematical model to estimate virus transmission as accurately as possible with limited data using more parameters.

### Model 6: Susceptible-Exposed-Symptomatic infectious-Hospitalized-Removed (SEIHR)

The SEIHR (Fig. 8) is a deterministic ODE model developed in [37] and was used to estimate the size of the outbreak and the reproduction number. They also evaluated the effects of different preventive measures. They used the daily data of confirmed cases in Daegu and North Gyeongsang (NGP), the main outbreak regions in Korea. According to the mathematical illustrations they found that if the rate of transmission decreases, the outbreak of the disease ends early; virus infection cases also decrease. They also suggested different social awareness activities such as wearing masks, social distancing and other intervention measures to reduce the fast transmission of the virus. They did not consider natural deaths and births, infections during latency and asymptomatic infections, or re-infected cases.

### Model 7: Susceptible-Exposed-Infectious-Hospitalized-Recovered-Dead (*Theta*-SEIHRD)

In [38], the authors proposed an extended compartmental model Theta-SEIHRD (Fig. 9) to investigate transmission dynamics of COVID-19 considering the known major characteristics of the virus. They studied the specific case of China including Hong-Kong, Macao, Taiwan and Mainland China and considered reported data that fit the parameters of the model that can be also useful to estimate the spread of COVID-19 in other countries. The presented model was adapted to the disease and is capable of estimating the progression of undetected and detected cases, deaths and the required number of beds needed in hospitals where the health problem is severe due to the outbreaks in territories, considering several different scenarios. In addition they calculated the basic reproduction number of COVID-19, which was 4.2250, but it fell to less than 1 after Feb 1, 2020, due to control measures. They also included in their model a new approach taking into account ‘Theta’, the fraction of total detected cases (infected), to study the importance of this fraction on the influence of COVID-19.

### Model 8: Susceptible Class-Exposed Class-Symptomatic and Infectious Class-Super Spreaders Class-Infectious but Asymptomatic Class-Hospitalized Class-Recovery Class-Fatality Class (SEIPAHRF)

The SEIPAHRF model (Fig. 10), developed in [23], gives a feasible approximation based on data in Wuhan, China, by studying some important aspects of COVID-19 transmission. They divided their model into eight important epidemiological compartments and illustrated the model mathematically with nonlinear ODEs. They investigated the sensitivity by considering the variations in its parameters. They also fitted the model with real data of daily confirmed cases and found the actual scenarios of the Wuhan outbreak. Interestingly they considered many parameters to quantify transmissibility and computed the basic reproduction number, a measure of virus spread based on Jacobian matrices. Limited data accessibility was one of the major limitations of this study.

### Model 9: Offspring Distribution

To estimate the level of over-dispersion in COVID-19 transmission and characterize the sustained transmission chains of human-to-human transmission, this model was introduced. Although only one research publication [40] was found, the mathematical implementation (see Overview section for more details) and statistical analysis of Offspring distribution looks powerful and should have the computational ability needed for a better estimation of moderate uncertainty levels with limited data resources. This was proven by a widely applicable Bayesian information criterion (WBIC). This model produced more accurate estimations than the Poisson branching process model. However, this model can only provide information about the lower boundary of the basic reproduction number RO(95% CrIs: R0 1.4–12) because of the marginal negative binomial distribution. Overall, the majority of model consistency and certainty relies on available homogeneous real data resources and imputed parameters by avoiding stochastic simulation.

**Table 2:** Mathematical models and their details

Basic model	Reference	Main findings	Strengths	Limitations
SIR	Vega 2020	Provides an overview to enhance awareness of COVID-19 disease trends	Investigated the effectiveness of social distancing considering both social contact and age structuring	Emphasizes quarantines only
		Effect of the quarantine in decreasing infection		
		Proposed extended lockdown		
	Zhong et al. 2020	Healthcaresystem could significantly shorten the outbreak period	Good ability to predict by historical datasuch as of the SARS 2003	Usedshort period data (two weeks)
		It could reduce one-half of the disease transmission.	It can also give a goodprediction of the limited COVID-19 data	
	Singh & Adhikari 2020	Accentuates the importance of both social contact and age structures	Estimates the contact structures	Insufficient data used in the asymptomatic case
		Social distancing is effective for controlling andmitigating the virus	Large-scale socialdistancing is effective	Themodel is not resolved spatially
SEIR	Kim et al. 2020a	Quantifying the school closure potential effect on the disease	Found schoolopening delay is effective	They did not considercross-population infection rise
		Considered isolation and behavior-changed susceptibleindividuals	The rate of child-to-childtransmission decreases	
	Lin et al. 2020	Captured thecourse of COVID-19 outbreaks	Considered: government actions	Considers small number ofconfirmed asymptotically infected transmission cases
		Computed the reportedratio and future trends	Individual behavioral responses	
		The method is applicable toother cities or other countries	Emigration oflarge portion of the people	
			Zoonotictransmission	
	Chang et al. 2020	Estimatedepidemic peak: In Wuhan and Hubei Province in the end of February2020	To estimate the epidemic trend, theyapplied phase-adjusted and region-adjusted mathematical model	Assumed diseases transmission evenlyacross homogeneous population
		Other regions in China on February 13, 2020		Total cases might beunderestimated as the existence of asymptomatic and super-spreadersinfectors
		Outbreaks would decrease in March and April inChina		Data lag might exist
	Kim et al. 2020b	Investigatedpattern of local transmission dynamics	Predicted the time of end of the corona outbreaks	Mortality rate was not included
		Found aper-capita infection transmissions rate 8.9 times higher in thelocal area (Daegu/Gyeongbuk) than nationwide (average).		
	Modnak & Wang 2019	The effects of infection latency and humanvaccination	Virus can spread from birdsto humans	Considerbi-linear incidence
			Human hosts	
	Tang et al. 2020a	Reproduction ratequantification for the evolution of interventions	Time-dependent contact and diagnose rates	Highlysensitive & depend upon available period data
	Prem et al. 2020	Physical distancing canreduce and delay the peak of the disease	Changes intransmission patterns decreased the number of cases in Wuhan	Individuals’ level heterogeneity is notcaptured in contacts
				Climatic factor is not included
				Large uncertainties over the estimation of reproductionand infectiousness duration
	Mandal et al. 2020	Found abasic reproduction rate of 1.5 the best case, and it reduces 62%cumulative incidence	Described rationalinterference to control the outbreaks	Used dataonly of airport entry individuals from China
		In worst case, basic reproductionrate is 4	Found potentialimpact of port entry screening	Ignoredtravelers from other countries
			A mitigation strategy ofsymptomatic cases	It may affectinfection duration; period of incubation and fatalityrate
	Kucharski et al. 2020	Estimated day-to-day reproduction number	Dynamics of transmission in Wuhan& risk of infections	Simple model
		Reproductionnumber declined from 2$\cdot$ 35 (95% CI 1 $\cdot$15-4$\cdot$ 77) to 1$\cdot$ 05(0$\cdot$41-2$\cdot$39) within one week		Transmission more homogeneous
		Found SARS-likevariations		
	Tang et al. 2020b	Calculatedthe effective daily ratio of reproduction	Used current revised data and information to estimatesoutbreaks trend	Needed to update parameters
		Re-estimated disease transmission risk		
		Evaluated theoutbreaks trend		
		Estimated disease peak phase		
	Yang and Wang 2020	Foundinfection transmission remain endemic	The reproduction rate was 4.25	Ecological, pathological andepidemiological aspects not clearly considered
		Long-term diseaseprevention and intervention programs are needed	Predicted the epidemic peak of the virusinfection	
M-SDI	Yin et al. 2020	Reproductionratio decreased from 1.7769 to approximately 0.97	Predictedthe multiple-information propagation trend	Used limiteddata for the estimation of parameters
		Public discussion peak was passed		
SUQC	Zhao & Chen 2020	Predictedtrends of transmission dynamics	Quantifying variables and parameters	Did not consider demographicfactors such as death
		Effects of quarantineor confirmation procedures on the disease	Able to provide guidance for other countries to controlthe outbreaks	
BHRP	Chen et al. 2020	Reproduction estimated fromreservoir to person and it is lower than from person to person	Used many parameters to quantify transmissibility	Used limited data
				Parameterassumptions
				Does not reflect the realresults
SEIHR	Choi & Ki et al. 2020	Estimated the size of theoutbreak and the reproduction number	Evaluated theeffects of different preventive measures	Did not consider natural deaths and births
				Latency andasymptomatic infections, and re-infected cases were notconsidered
SEIHRD	Ivorra et al. 2020	Calculatedbasic reproduction number	Estimated basic reproduction rate and percentage ofundetected cases	Spatial distributionwithin the territory is omitted
		The effective reproductiondecreases due to the different control measures taken		Between-countrytransmission was not considered
				Officially releaseddata was not of high quality due to severaluncertainties.
SEIPAHRF	Ndairou et al. 2020	Investigated thesensitivity by considering the variations of its parameters	Considered many parameters to quantify transmissibilityand computed the basic reproduction number	Limited datawere studied
Offspring distributions	Endo et al. 2020	Better estimation of moderate uncertaintylevels with limited data resources	Moderate uncertainty levels	Highly over dispersed due toa very small fraction of individuals
		Provided a lowerboundary of the basic reproduction number		

## Observable Behavior of the Different Models

Next, we address some key observable behaviors of different models within the context of COVID-19 mathematical modeling, data sources, applied methods, used parameters and result prediction. The base principle of this subsection is to give the overall effectiveness of the specific model in the context of the COVID-19 pandemic. We also describe these behaviors in Table 2. In addition, Table 3 highlights the important comparisons between the models.

### Model 1: Susceptible-Infectious-Removed (SIR)

- SIR is the basic compartmental model used to assess the pandemic size, and transmission peak and to predict the trends of the current COVID-19.
- Identified effectiveness of lockdowns/quarantines and different social distancing measures through parameter estimation.
- This model can be used for disease mitigation policies according to numerical illustrations in the context of COVID-19.

### Model 2: Susceptible-Exposed-Infectious-Removed (SEIR)

- The SEIR model is also applicable to assess the transmission dynamics of contagious disease such as COVID-19.
- In SEIR the exposed compartment as well as an additionally adopted parameter (the movement from the exposed compartment to the infected compartment) were used to describe the transmission dynamics of exposed individuals in the context of COVID-19.

**Table 3:** Head-to-head comparison between the models: the first column indicates the specific model and illustrates the model to be compared with respect to the models presented in the first row. In other words, this is an antisymmetric information matrix other than the first row and column. [**E**-exposed class, **I**-infectious, **Isi**-symptomatic infectious, **U**-unquarantined infected class, **Q**-quarantine infected class, **C**-confirmed infected class, **D**-discussing class, **Im**-immune class, **SI**-symptomatic infected, **AI**-asymptomatic infected, **H**-hospitalized, **SS**-super spreader class, **F**-fatality class, **Stm**-secondary transmission]

	SIR	SEIR	M-SDI	SUQC	BHRP
**SIR**		$$\begin{array}{l} \hbox {E absent}\\ \hbox { Fewer parameters}\end{array}$$	$$\begin{array}{l} \hbox {D and I absent }\\ \hbox { Different parameters}\end{array}$$	$$\begin{array}{l} \hbox {U, Q and C are absent}\\ \hbox {Different parameters}\end{array}$$	$$\begin{array}{l} \hbox {E, SI and AI are absent}\\ \hbox {Differently parameterize}\end{array}$$
**SEIR**	$$\begin{array}{l}\hbox { E added}\\ \hbox { More parameter appended}\end{array}$$		$$\begin{array}{l} \hbox {E, I and R are different} \\ \hbox {Different parameter approximation}\end{array}$$	E, I and R are different from U, Q and C	$$\begin{array}{l} \hbox {I is different from SI and AI }\\ \hbox {Simple than RP}\end{array}$$
**M-SDI**	$$\begin{array}{l}\hbox { I and R absent}\\ \hbox {Public opinion based}\end{array}$$	$$\begin{array}{l} \hbox {E, I and R absent}\\ \hbox {Parameterized public opinion}\end{array}$$		$$\begin{array}{l} \hbox {U, Q and C are absent}\\ \hbox {Multiple-information based}\end{array}$$	$$\begin{array}{l} \hbox {Different approach }\\ \hbox {Public opinion data}\end{array}$$
**SUQC**	$$\begin{array}{l} \hbox {U, Q and C are different}\\ \hbox {R absent}\end{array}$$	$$\begin{array}{l} \hbox {U different from E }\\ \hbox {R absent}\end{array}$$	Different approach		$$\begin{array}{l} \hbox {E and R absent}\\ \hbox {U, Q and C appended}\end{array}$$
**BHRP**	$$\begin{array}{l} \hbox {E appended}\\ \hbox {SI and AI different from I}\end{array}$$	SI and AI different from I	Different approach	$$\begin{array}{l} \hbox {E and R appended}\\ \hbox {SI and AI different from U and Q}\end{array}$$	
**SEIHR**	E and H appended	H appended	Different model	$$\begin{array}{l} \hbox {E, H, R appended}\\ \hbox { I not same as U, Q and C}\end{array}$$	$$\begin{array}{l} \hbox {H appended}\\ \hbox {Isi different from SI and AI}\end{array}$$
**Theta-SEIHRD**	$$\begin{array}{l} \hbox {H, R and D appended }\\ \hbox {SI(=I) is different}\end{array}$$	$$\begin{array}{l} \hbox {H appended}\\ \hbox {SI(=I) is different}\end{array}$$	Different model	$$\begin{array}{l} \hbox {I different}\\ \hbox {H, R and D appended}\end{array}$$	$$\begin{array}{l} \hbox {I different }\\ \hbox {H appended}\end{array}$$
**SEIPAHRF**	$$\begin{array}{l}\hbox { Isi(=I) different from I }\\ \hbox {E, P, A and H appended}\end{array}$$	$$\begin{array}{l} \hbox {Isi(=I) different from I }\\ \hbox {P, A and H appended}\end{array}$$	Different model	E, I, P, A and H differently appended	P and A appended
**Offspring distributions**	Different model Assumed Stm	Different model Assumed Stm	$$\begin{array}{l} \hbox {Different model}\\ \hbox {Assumed Stm}\end{array}$$	$$\begin{array}{l} \hbox {Different model}\\ \hbox {Assumed Stm}\end{array}$$	$$\begin{array}{l} \hbox {Different model}\\ \hbox {Assumed Stm}\end{array}$$

	SEIHR	Theta-SEIHRD	SEIPAHRF	Offspring distributions	
**SIR**	$$\begin{array}{l} \hbox {E and H absent}\\ \hbox {Fewer parameters}\end{array}$$	$$\begin{array}{l} \textit{Theta as fraction and E and H absent}\\ \textit{Fewer parameters}\end{array}$$	$$\begin{array}{l} \hbox {E,SI, AI,SS,H and F absent}\\ \hbox { Fewer parameters}\end{array}$$	Different Approach	
**SEIR**	$$\begin{array}{l} \hbox {I is different from SI and H }\\ \hbox {Parameters estimations different}\end{array}$$	$$\begin{array}{l} \hbox {H is absent}\\ \hbox {Parameter approximation different}\end{array}$$	$$\begin{array}{l} \hbox {H absent}\\ \hbox {I in simplest form }\\ \hbox {Parameter representation different}\end{array}$$	Different Approach	
**M-SDI**		Different approach	Analyzed users propagation	Different Approach	
**SUQC**	$$\begin{array}{l} \hbox {E, H and R absent}\\ \hbox {U, Q and C appended}\end{array}$$	$$\begin{array}{l} \hbox {E, H, R and D absent }\\ \hbox {U, Q, C appended}\end{array}$$	$$\begin{array}{l} \hbox {E, I, P, A, H, R and F are different and absent }\\ \hbox {U, Q and C appended}\end{array}$$	Different Approach	
**BHRP**	$$\begin{array}{l} \hbox {SI and AI are different from I}\\ \hbox {H absent}\end{array}$$	$$\begin{array}{l} \hbox {SI and AI are different from I} \\ \hbox {H absent}\\ \hbox {Fraction Theta absent}\end{array}$$	$$\begin{array}{l} \hbox {SI and AI are different }\\ \hbox {H absent}\end{array}$$	Different Model	
**SEIHR**		Fraction Theta is absent	$$\begin{array}{l} \hbox {Isi(=I) is same as SI(=I) }\\ \hbox {P and A absent}\end{array}$$	Different Model	
**Theta-SEIHRD**	I different from Isi		$$\begin{array}{l} \hbox {I different }\\ \hbox { P, A absent}\end{array}$$	Different Model	
**SEIPAHRF**	P and A appended	$$\begin{array}{l} \hbox {Fraction Theta absent }\\ \hbox {P A appended}\end{array}$$		Different Model	
**Offspring distributions**	$$\begin{array}{l} \hbox {Different model}\\ \hbox {Assumed Stm}\end{array}$$	$$\begin{array}{l} \hbox {Different model }\\ \hbox {Assumed Stm}\end{array}$$	$$\begin{array}{l} \hbox {Different model }\\ \hbox {Assumed Stm}\end{array}$$		

### Model 3: Multiple-Information Susceptible-Discussing-Immune (M-SDI)

- This approach is different from the studied models in this research as the model is developed based on information propagation on social media especially on the topic of public discussions in the context of COVID-19.
- In this study, the authors focused on the characteristics of users choosing to discuss certain topics related to COVID-19.
- To predict public discussion trends they approximated parameters using the LS method and estimated the public discussion peak by calculating the reproduction ratio.

### Model 4: Susceptible-Unquarantined Infected-Quarantine Infected-Confirmed Infected (SUQC)

- This model was developed in [34] to describe the COVID-19 transmission dynamics.
- The interference effects of control measures are especially parameterized by analyzing the disease outbreak.
- This model was adapted to daily released confirmed case data to analyze the outbreaks in Hubei, Wuhan, and four other first-tier cities in China.
- They considered multiple characteristics to predict the transmission trends.

### Model 5: Bats-Hosts-Reservoir-People (BHRP)

- The BHRP model might be an advanced version of both the SIR and SEIR models.
- The BHRP mathematical computational part involves many differential equations.
- The most challenging part is the estimation of many parameters.
- More data are needed to better understand the transmission dynamics in the context of COVID-19.

### Model 6: Susceptible-Exposed-Symptomatic Infectious-Hospitalized-Removed (SEIHR)

- The SEIHR model estimates the size of the outbreak and the reproduction number, and they evaluate the effects of different preventive measures.
- Results demonstrated that wearing masks, maintaining social distance and other intervention measures can reduce the fast transmission of the COVID-19 virus.
- In this study, they did not consider natural deaths and births, infections during latency and asymptomatic infections, and re-infected cases.

### Model 7: Susceptible-Exposed-Infectious-Hospitalized-Recovered-Dead (*Theta*-SEIHRD)

- Theta-SEIHRD was developed to assess the dynamics of COVID-19 by considering its major known characteristics.
- In this method, the authors added ‘Theta’, the fraction of total detected cases (infected) to study the importance of this fraction on the influence of COVID-19.
- They fitted and estimated many parameters with reported data in the model which could be useful to estimate the spread of COVID-19 in some other countries.
- The reported data that used was homogeneously distributed and weak in quality (uncertainty in both undetected infections and a number of characteristics of the virus).

### Model 8: Susceptible Class-Exposed Class-Symptomatic and Infectious Class-Super Spreaders Class-Infectious but Asymptomatic Class-Hospitalized Class-Recovery Class-Fatality class (SEIPAHRF)

- The SEIPAHRF model gives a feasible approximation by studying different important aspects of COVID-19 transmission.
- The model was illustrated mathematically with nonlinear ODEs, and sensitivity was investigated by considering the variations of its parameters.
- They also fitted the model with real, daily confirmed case data and considered many parameters to quantify transmissibility.
- According to the theoretical findings and numerical illustrations, the model was well adapted to the actual data and it reflected the real scenarios in Wuhan, China.

### Model 9: Offspring Distribution

- Offspring Distribution performs better for uniform and homogeneous data.
- Computationally, this model is powerful.
- It might be challenging to include additional parameters and distinguish stages that are quite easily applicable and identifiable in other mentioned models mentioned above.

Most of these studies did not consider natural deaths and births although demographic factors (birth, death, emigration and immigration) need to be added to the compartmental models for more realistic outcomes. Moreover, for the M-SDI model, the authors used public opinion data, which is different from other models. The Theta-SEIHRD model used ‘Theta’ the fraction of total detected cases (infected) to assess the significance of this fraction on the influence of COVID-19. In the SUQC model, the authors parameterized the interference effects of control measures by characterizing multiple aspects to predict transmission trends. In addition, the SEIHR model demonstrated that wearing masks, maintaining social distances and other intervention measures can reduce the fast transmission of the virus by calculating the effects of different preventive measures. Furthermore, it was found that the offspring distribution model performed well with uniform and homogeneous data. The SEIPAHRF model was fitted by considering many parameters to quantify transmissibility. Subsequently, more data are needed for a better understanding of the transmission dynamics in the context of COVID-19 in the BHRP model.

Accordingly, using more parameters and adding more stages make a model more complex and computationally challenging. Using original values and data collected from the field work certainly moves a model toward an accurate prediction of the near future. More complex models and methods in the future might handle these upcoming challenges.

## Other Relative Mathematical Models and Studies

Our review was prepared by following the most commonly used mathematical models based on original data collected from reliable resources. However, there are a few other mathematical models available for data analysis of COVID-19 transmission dynamics analysis. Here, we present a short overview of some recent work.

### Fractional Order and Relative Studies

Very recently, a few other interesting mathematical models have been proposed such as fractional-order models [82, 83], which are used for simulation dynamics [83], qualitative analysis [53] and numerical analysis [82]. A new Caputo-Fabrizio fractional order was also proposed for the SEIASqEqHR model for COVID-19 transmission with a genetic-algorithm-based control strategy [49]. Similar studies were done by a Nigerian research group [82] and a Saudi Arabian group [83]. Computational and theoretical modeling of the transmission dynamics of COVID-19 was also proposed under the Mittag-Leffler power law considering a fractional-order epidemic model [50]. In another study, a fractional order epidemic model with numerical simulation was proposed for global stability analysis using the Lyapunov candidate function [48]. Another study introduced a Haar wavelet collocation approach for a solution to the fractional-order COVID-19 model using the Caputo derivative [52]. A numeric simulation of fractional order was performed on the basis of the Wuhan COVID-19 case study [54]. However, fractional-order model has also been applied using the real cases reported in Saudi Arabia [55], USA [56], Pakistan [58] and Nigeria [57].

### Simulation-Based Studies

A simulation-based study proposed the dispersal effect [84] to understand the dynamics of the disease by boundedness and non-negativity of solutions. Another simulation-based SQIR-type model under the nonlinear saturated incidence rate was also proposed [39]. Another simulation-based study was used for the age-structured modeling of COVID-19 in the USA, UAE and Algeria [51]. Another study provided guidelines on how to run the analysis for COVID-19 with optimal control, stability and simulations [85]. Another simulation-based study presented a global stability analysis [86].

## Challenges and Future Research Directions

Symptoms of COVID-19 can vary by area, age group and even individual because of its mutations. Therefore, it will be extremely challenging to predict future research directions. Here, we present a list of challenges to be faced by the existing models and possible ways mitigating them.

### Data Size

COVID-19 data are increasing day-by-day and the data storage system is becoming rich. This increases the possibility of finding the most accurate model in the near future. In addition, these data encourage researchers to consider even more complex models by mining the available information. However, it is extremely challenging to handle such a high amount of data and to select the right model for accurate future prediction regarding COVID-19 transmission. More computational power (e.g., supercomputers) will be required for analysis.

### Parameter Estimation

How good a model is depends on the model parameters used. Since a large dataset invites researchers to implement more complex models, there is always an important issue regarding parameter estimation. Complex models often use simulated data for unknown parameter estimation and become less accurate. In other words, it is always expected to use real data for parameter estimation to predict the real virus transmission rate. Less complex models should be chosen in the case of limited data.

### Survival & Lost Dynamics

Most COVID-19 studies compute transmission of COVID-19 following only the nature of survival dynamics. However, lost dynamics (where, when and how people die) might also be valuable information for future prediction. Just computing the results of COVID-19 death might not help us to understand its true dynamics. Combination of survival and lost dynamics might provide us a true picture of the reality.

### Undetected Cases/Self-quarantine

Although the COVID-19 data storage/backup system is becoming richer by the day, people worldwide, with or without symptoms, are afraid of being tested for COVID-19 and having to self-quarantine with [38, 87]. The actual number of people who need to quarantine might never be known unless a safe and remote personal testing kit is developed and people testing positive are encouraged people not to hide that information from the proper authorities. For mathematical modeling, a quarantine might be a challenging stage regarding parameter estimation, which might impact in the final transmission rate of COVID-19.

### Reinfection

Scientists cannot guarantee that one person cannot catch COVID-19 twice even though increased immunity was observed in a patient after recovery. An online news portal reported that a Chinese group found evidence of it being caught twice in a monkey study. Thus, the possibility of reinfection might not be precluded. The model researchers might not be able to separate the already infected from upcoming analyses. Most recently, several types of vaccines (e.g., mRNA) have been introduced and priority has been given to medical workers and the elderly. However, it has been heard that very few people are getting infected after vaccination. It is predicted that COVID-19 might continue flu like virus and people might need to be vaccinated after a certain period each year.

### Mutations

Human civilization may face enormous challenge near new future mutations, specifically in glycoproteins [88]. Scientists have already spotted thousands of generic material changes in the coronavirus and its mutations worldwide. However, not all of the mutations infect all parts of the world. For country- or region-specific mathematical modeling, it is also important to include the number of mutations of the virus (if possible). This information can be investigated for weather/area/temperature-based virus transmission. A new strain called SARS-CoV-2 VUI 202012/01 or “B.1.1.7.” has caught attention which was first discovered in the southeast UK in September 2020. It might be more contagious than other available strains (approximately 70% faster than the usual one) and is gradually spreading other countries. This strain of virus might infect any age people including children.

### Multiple Model Implementation

Which model fits well depends on the available data and the goal of the analysis. The researchers’ main goal is to implement a specific model or an improved version of the model to investigate virus transmission. However, applying different models (e.g., SIR and SEIR) in the same study might provide the range and center of true expected virus transmissions. In addition, limitations on parameters between different models might be overcome or minimized.

### Upcoming Waves

Human civilization has learned about COVID-19 and passed the second attack (often called second wave) almost all around the world [89]. Almost every countries have made wearing a mask mandatory in all indoor public areas. Currently, 3rd wave is running following new variants and people might still be infected and the data will be stored. We are not sure about how many waves will come in future. For mathematical modeling, researchers should consider this issue and add few new stage ( following the number of waves) of the COVID-19 transmission with new parameters based on the data information. This model might help us to predict the timing and strength of upcoming waves.

Next, we identify some of the possible future directions of mathematical modeling.

### Decision Support System (DSS)

So far, no guaranteed antiviral treatment has been recommended regarding COVID-19 [90]. The only way to fight against this contagious disease is to emphasize preventive healthcare systems and implement necessary rules and regulations that depend on the spreading nature of virus transmission. Here, a decision support system (DSS) [91] can play a very important role by having priority guidelines (e.g., social distancing/lockdown) administered by national and international authorities in an effective and efficient way. The outcome of mathematical modeling can be plugged into this DSS system. However, the necessary supporting information can be collected from the corresponding authority.

### Artificial Intelligence (AI)

Most AI implementations for COVID-19 analysis rely on a convolutional neural network following X-ray and CT images. However datasets are also available for aggregated case reports, management strategies, the healthcare workforce, the demography and the mobility during the outbreak. Interestingly, both mathematical modeling and AI have shown their ability as reliable tools to fight against the COVID-19 pandemic [92]. These tools altogether might lead to the new insights on this pandemic. Very recently, a deep learning algorithm was introduced for the modeling and forecasting of COVID-19 in the five worst affected states of India [93].

### Other Implementable Mathematical Methods

The field of mathematics has been expanding over time with new methods and implementations. Most COVID-19 studies have applied ordinary differential equations (ODEs) by adding suitable parameters. However, partial differential equations (PDEs) can also be useful in similar studies when other parameters do not change with respect to a specific parameter. To solve the equations (e.g., ODEs) with higher numbers of unknown variables (known as parameters in COVID-19 studies), Galois theory [94] can be used. However, to investigate the specific effects of similarities between different areas or countries of COVID-19, Polya’s theorem [95] can be applied. Fuzzy logic can also be used as it is able to handle uncertainties by considering several membership functions based on the transmission dynamics trends data of COVID-19.

## Discussion and Conclusion

To identify and investigate the spread of COVID-19 and to save human civilization, mathematical modeling might play an important role. In this review, we addressed a number of mathematical models and how different methods can be applied to understand the comprehensive behavior involved in COVID-19 transmission dynamics. We also considered several other key issues such as applied parameters, different data sources and predicted results. Applying different models and methods under the same investigation might reflect the model variability and provide a boundary signature about the future situation.

Besides our findings, continuing research on COVID-19 might not be able to save human civilization unless proposed rules and regulations are strictly implemented considering the stage of the pandemic of that area/region. A DSS might help the local authority by providing necessary guidelines following the mathematical modeling of transmission dynamics [91]. Everyone in the locality must follow the directions as well as they can. However, some people are already at a high risk due to job condition (e.g., nurses and doctors), medical condition (e.g., heart disease, lung disease, diabetes, or cancer) and age [96] (e.g., high risk for persons aged (65+)). Special monitoring systems and better facilities are in demand to protect them. In the bigger context, the world is united against COVID-19 to stop its transmission.

How useful a model/method is depends mostly on the availability of a sufficient amount of real data and the usage of specific parameters reflecting the real value. Importantly, a more elaborate dataset (containing more information) might provide valuable information for model prediction. So far, a text-based spreadsheet and medical imaging data [97] are available. Surprisingly, a COVID-19 mouse is also already available for research [98]. New insights on COVID-19 might be found through rodent behavioral experiment [99–103], brain imaging [99–104], advanced computational image analysis [100, 103, 105] and cellular level gene expression [106]. Such imaging and gene data can be helpful for mathematical modeling and data analysis as to investigate long-term future effects, to make predictions and prevent COVID-19 transmission.

The number of daily new cases is gradually going down, and a number of vaccines (e.g., Pfizer-BioNTech, Astrazeneca, Moderna and J & J) are already administered. People are more aware than they were in the first wave and all around the world are receiving vaccines with the priority being on the elderly and medical workers. However, several new variants (e.g., the UK/Kent variant: B.1.1.7, the Indian Variant (B.1.617.2), the South African variant (B.1.351) and the Brazilian variant (P.1)) have emerged. The current challenges for these new variants are to determine how fast they spread. Mathematical modeling can again be used to identify the transmission behavior of these new variants.

## Data Availability

We do not have any data, materials, codes, supplementary materials or supporting information available anywhere else other than this manuscript.

## Abbreviations

BHRP: Bats-Hosts-Reservoir-People
DSS: Decision support system
M-SDI: Multiple-information susceptible-discussing-immune
SIR: Susceptible-Infectious-Removed
SEIR: Susceptible-Exposed-Infectious-Removed
SUQC: Susceptible-unquarantined infected-quarantine infected-confirmed infected
SEIHR: Susceptible-Exposed-Symptomatic infectious-Hospitalized-Removed
Theta-SEIHRD: Susceptible-Exposed-Infectious-Hospitalized-Recovered-Dead
SEIPAHRF: Susceptible class-exposed class-symptomatic and infectious class-super spreaders class-infectious but asymptomatic class - hospitalized class-recovery class-fatality class
